# The expansion and diversity of the *CYP75* gene family in Vitaceae

**DOI:** 10.7717/peerj.12174

**Published:** 2021-09-15

**Authors:** Yang Xiao, Jun Wen, Ran Meng, Ying Meng, Qiang Zhou, Ze-Long Nie

**Affiliations:** 1 Key Laboratory of Plant Resources Conservation and Utilization, College of Biological Resources and Environmental Sciences, Jishou University, Jishou, Hunan, China; 2 Department of Botany, National Museum of Natural History, Smithsonian Institution, Washington, D.C., U.S.A.

**Keywords:** *CYP75*, Gene family, Vitaceae, Gene duplication

## Abstract

The *CYP75* gene family plays an important role in flavonoid biosynthesis in plants. Little is known about the evolution of the gene family within the grape family. Here, we extracted the *CYP75* genes from transcriptome data of 15 grape species and 36 representative genomes from other plants to explore the evolutionary history of the *CYP75* gene family in Vitaceae. The structure of the CYP75 protein sequences is highly conserved with the variation mainly occurring in the N terminal and the middle region. The evolutionary analyses suggested classifying the *CYP75* gene family into three groups in Vitaceae, namely Vitaceae A1, Vitaceae A2 and Vitaceae B. The Vitaceae A1 and A2 belong to the *CYP75A* subfamily and the Vitaceae B belongs to the *CYP75B* subfamily. Within the Vitaceae A1, most Vitaceae taxa present only one copy of the CYP75A protein sequence except for *Vitis vinifera* with a high number of sequences, which might have originated through recent gene duplications after its split from the other species. Vitaceae A2 contain only CYP75A sequences from Vitaceae sister to one from *Camellia sinensis*, probably representing a relict lineage. The CYP75B proteins were found to be dominated in Vitaceae and other angiosperms. Our results provide important insights into understanding the evolutionary history of the *CYP75* gene family in Vitaceae and other angiosperms.

## Introduction

Gene duplication can provide raw genetic material for biological evolution and may arise through whole-genome duplication (WGD), transposon-mediated duplication, segmental duplication, tandem duplication, or retroposition (Panchy, Lehti-Shiu & Shiu, 2016; Zhang, 2003). Among them, WGD is considered a major mechanism for the evolution of morphological and physiological diversity (Paterson et al., 2010; Soltis et al., 2009). Because plants are commonly diploidized polyploids with numerous duplicated chromosomal blocks within their genomes, segmental duplications occur frequently in plants (Cannon et al., 2004). Unlike segmental duplication, an important character of tandem duplication is multiple members of a gene family occurring within the same intergenic region or in neighboring intergenic regions (Ramamoorthy et al., 2008). Gene duplications play an important role in the evolution of multi-gene family (Horan et al., 2005; Maere et al., 2005; Zhang, 2003). Segmental and tandem duplication are important for the generation and maintenance of gene families (Cannon et al., 2004). After gene duplication, most duplicated genes are lost, and a few duplicated genes are retained (Lynch & Conery, 2000; Zhang, 2003).

Cytochrome P450 (*CYP*) superfamily is the largest plant enzyme family in plant metabolism and accounts for about 1% of all gene annotations of each species (Mizutani & Ohta, 2010; Nelson & Werck-Reichhart, 2011). *CYP* genes play important roles in plant development and defense, through their involvement in the synthesis of secondary metabolites, the regulation of plant hormone metabolism, and phytoalexin biosynthesis (Jun, Wang & Guo, 2015). *CYP* genes with an amino acid sequence identity greater than 40% are grouped into a gene family, while sequence identity greater than 55% are further divided into subfamilies (Nelson et al., 1996). *CYP* genes in land plants contain 11 clans classified into two clusters: single-family clans (CYP51, CYP74, CYP97, CYP710, CYP711, CYP727, CYP746) and multi-family clans (CYP71, CYP72, CYP85, CYP86) (Nelson & Werck-Reichhart, 2011).

CYP75 for flavonoid biosynthetic enzymes belongs to clan71 (Nelson & Werck-Reichhart, 2011), which involves in the biosynthesis of the majority of plant secondary metabolites in adaptation to biotic and abiotic stress (Morant et al., 2007). The *CYP75* gene family is composed of two subfamilies, *i.e.,* *CYP75A* (flavonoid 3′,5′-hydroxylase, *F3′5′H*), *CYP75A*) and *CYP75B* (flavonoid 3′-hydroxylase, *F3′H*), which catalyze the hydroxylation of the B-ring of flavonoids, an important biosynthesize process in cyanidin and delphinidin synthesis and the precursors of blue and red anthocyanins (Tanaka, 2006). The hydroxylation patterns play a role in the determination of flower and fruit color (Tanaka & Brugliera, 2013).

Both *CYP75A* and *CYP75B* genes were first identified from petunia (Brugliera et al., 1999; Holton et al., 1993) and then from *Arabidopsis thaliana* (Schoenbohm et al., 2000), *Glycine max* (Toda et al., 2002), *Vitis vinifera* (Castellarin et al., 2006), *Solanum lycopersicum* (Olsen et al., 2010), *Epimedium sagittatum* (Huang, Sun & Wang, 2012), *Camellia sinensis* (Wang et al., 2014), *Pohlia nutans* (Liu, Ju & Xia, 2014), *Delphinium zalil* (Miyahara et al., 2016), and *Hordeum vulgare* (Vikhorev, Strygina & Khlestkina, 2019). In many plants including *Arabidopsis thaliana*, *Dianthus caryophyllus*, and rose, there is an absence of delphinidin-based anthocyanins, indicating these plants lost *CYP75A* genes during the evolution (Tanaka & Brugliera, 2013; Tanaka & Brugliera, 2014). In the Asteraceae, some *F3*′5′*H* genes belong to the *CYP75B* genes rather than *CYP75A* genes, indicating the independent evolution of an Asteraceae-specific *F3*′5′*H* (Seitz et al., 2006). Similarly, rice *CYP75B4* also functions as an *F3*′5′*H* (Lam, Liu & Lo, 2015). These species had lost *F3*′5′*H* genes from *CYP75A* subfamily and then probably reacquired the *F3*′5′*H* genes from *CYP75B* subfamily by gene duplication and neo-functionalization (Seitz et al., 2006; Tanaka & Brugliera, 2014). This hypothesis was supported by the exchange of *F3′H* and *F3*′5′*H* activities through several amino acid substitutions (Seitz, Ameres & Forkmann, 2007).

Phylogenetic analysis of known *CYP75* genes suggested that the *F3*′*H* and *F3*′5′*H* functions were established and split before the divergence of gymnosperms and angiosperms (Seitz et al., 2006; Ueyama et al., 2002). Although previous studies have shown that the *CYP75* genes appear in angiosperms and gymnosperms but not in bryophytes and ferns (Nelson & Werck-Reichhart, 2011), some evidence suggests that bryophytes and ferns also have genes function similarly as the *CYP75* family (Liu, Ju & Xia, 2014; Zhang et al., 2019). Monocot *CYP75B* genes were split into two independent lineages (Class 1 and Class 2) after gene duplication in the common ancestor (Jia et al., 2019).

For *Vitis vinifera*, the *CYP75* gene family has attracted the attention of many researchers. This is mainly because the *CYP75* gene family plays an important role in the determination of fruit color and flavor (Castellarin et al., 2006; Falginella et al., 2010; Jeong et al., 2006). *Vitis vinifera* genome contains two copies of *CYP75B* (*F3′H*) genes and 16 copies of *CYP75A* (*F3′5′H*) genes (Falginella et al., 2010). The 16 copies of *CYP75A* genes are located on two chromosomes, 15 copies on chromosome 6 and one on chromosome 8. The 15 copies were recently generated by gene duplication within the Vitaceae after the separation from other dicot lineages, contributing to the abundance of different anthocyanins in berry skin (Falginella et al., 2010). *Vitis vinifera* belongs to Vitaceae, which consists of approximately 16 genera and approximately 950 species primarily distributed in tropical regions, with a few genera in temperate regions (Soejima & Wen, 2006; Wen et al., 2018). With a transcriptome approach, the major clades were resolved with highly significant statistical support (Wen et al., 2013). However, to date, the evolutionary history of the *CYP75* gene family for the other Vitaceae taxa remains elusive.

In this paper, we retrieved and identified the *CYP75* genes from the transcriptomes of Vitaceae and other plant representative genomes. Phylogenetic analyses were performed to identify and compare the *CYP75* gene family in Vitaceae in the framework of seed plants. The conserved motifs and domain of CYP75 proteins were examined. We also checked the lineage-specific branch evolutionary pressure by selective pressure analysis. Our results will provide important evidence for understanding the evolutionary history of Vitaceae *CYP75* gene family after gene duplication as well as insights into the evolution of the *CYP75* family in angiosperms as a whole.

## Materials & Methods

### Data sources

To investigate the evolution of the *CYP75* gene family in Vitaceae, transcriptomic data of 15 species of Vitaceae were used from our previous work (Wen et al., 2013). The transcriptome de novo assembly was re-performed using Trinity v2.4.0 (Haas et al., 2013). To investigate the evolutionary history in a broad framework, representative genomes of other land plants were selected from NCBI databases or other sources (Table S1). The samples selected included one bryophyte, three gymnosperms, and 32 angiosperms. In particular, the proteome of *Abies alba* was obtained from the *Abies alba* genome project (Mosca et al., 2019); the proteome of *Ginkgo biloba* was obtained from the *Ginkgo biloba* genome project (Guan et al., 2016); and the proteome of *Gnetum montanum* was obtained from the *Gnetum montanum* genome project (Wan et al., 2018). *Amborella trichopoda* and *Aquilegia coerulea* represent basal angiosperm and basal eudicot, respectively. Bryophyte species *Physcomitrella patens* and three gymnosperm species were selected as the remote outgroup to root our tree topology.

### Gene identification

To identify the *CYP75* gene family numbers in Vitaceae and other plants, the protein sequences of five *CYP75A* genes (AAP31058, AB078781, AJ011862, Z22544, BAA03439, BAA03440) and three *CYP75B* genes (AY117551, BAD00189, AF155332) were downloaded from NCBI as a query. The eight known CYP75 protein sequences were used to identify candidate *CYP75* genes from Vitaceae transcriptomes and other genomes *via* tBlastN program. Candidate genes were retained at thresholds of E<e-20 and amino acid identity >50% (Falginella et al., 2010). The hidden Markov model-based HMMER software was used to examine all protein sequences of the candidate *CYP75* genes. The proteins containing the P450 domains were considered as CYP75 proteins and included in this study.

### Phylogenetic analyses

Multiple sequence alignments were performed by Mafft (Katoh, 2002) with default parameters, which were used for phylogenetic analyses. The result of the alignment was adjusted manually in PhyDE. Phylogenetic analyses were conducted by using the Maximum Likelihood (ML) method, and the ML tree was constructed by using RAxML 8.2.10 (Stamatakis, 2014). The best model (JTT+G+I) was determined by using the Akaike Information Criterion (AIC) and the Bayesian Information Criterion (BIC) based on modeltest-ng-0.1.3 (Darriba et al., 2020). We used the rapid bootstrapping algorithm to perform 1000 bootstrap replicates. ML bootstrap values of each node were visualized using FigTree 1.4 (http://tree.bio.ed.ac.uk/software/figtree/).

### Identification of *CYP75* protein motifs

To unveil motifs variation among related proteins within the *CYP75* gene family in Vitaceae and other plants, the MEME motif search tool (Bailey et al., 2009) was employed to identify conserved motifs in the 135 putative CYP75 protein sequences. The optimized parameters of MEME were employed as follows: the maximum number of motifs to be found was set at 25; the optimum width of each motif, between 6 and 50 residues. All CYP75 protein sequences were used to compare the differences in each clade.

### Detection of positive selection

To explore if the *CYP75* gene family was subjected to selective pressure in angiosperms, we investigated variable selective pressures of *CYP75* genes in angiosperms, specifically between monocots and dicots in each gene subfamily. A reduced ML tree was used to estimate the selection pressure in the *CYP75* gene family of angiosperms. Sequences from Vitaceae transcriptomes were excluded to avoid potential taxon biases. The protein sequence alignments and the relative cDNA sequences were converted into corresponding codon alignments by using the PAL2NAL (Mikita, David & Peer, 2006). The branch model was used to evaluate the *ω* values (*ω* = dN/dS) with the codeml program from the PAML 4.9 package (Yang, 1997). The model 0 (all branches are fixed to the same *ω* value) and the model 2 (some branches have different *ω* values) to detect the selection pressures in different branches. LRT (likelihood ratio test) was conducted to determine whether there was statistically significant heterogeneity between the two models and whether *ω* ratios were different.

## RESULTS

### Identification of *CYP75* gene family and phylogenetic analysis

A total of 135 *CYP75* genes were obtained including 36 *CYP75* genes from Vitaceae and 99 *CYP75* genes from other 36 plant genomes (Table S1). *CYP75* genes were not found in *Cissus microcarpa* and some Vitaceae *CYP75* genes include incomplete protein sequences with a shorter sequence length (Table 1). All protein sequences of putative 135 *CYP75* genes were checked to confirm the presence of the P450 domains.

**Figure 1:**
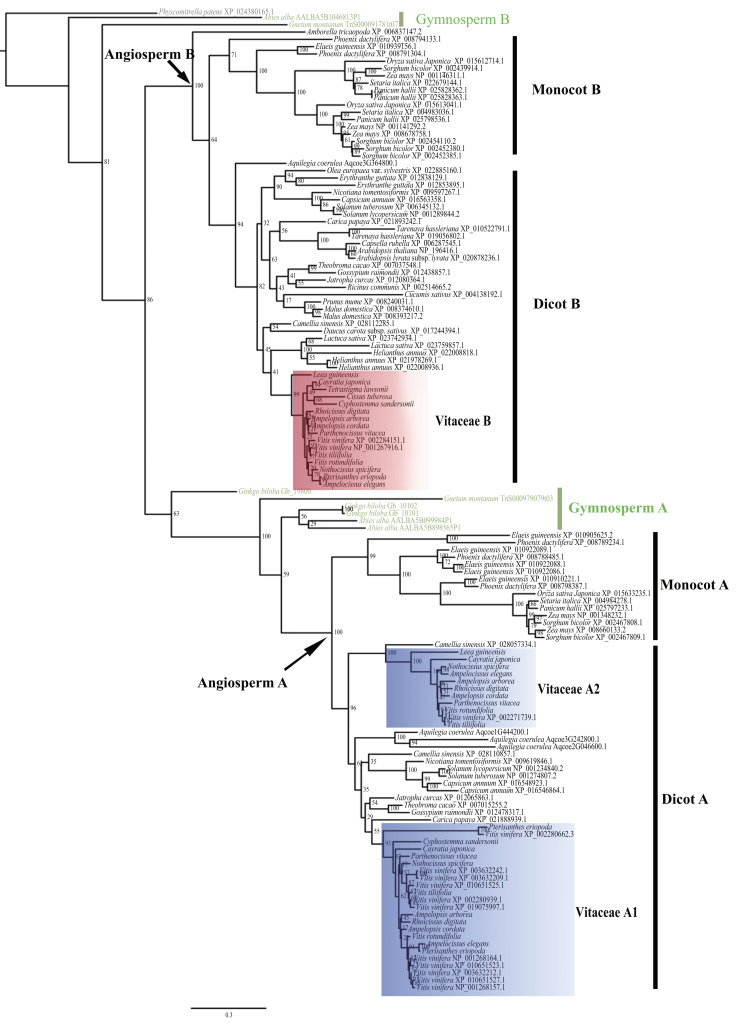
Phylogenetic relationships of *CYP75* gene family in Vitaceae.

The evolutionary relationship of the 135 CYP75 proteins was shown in Fig. 1. Based on the tree topology and taxonomic distribution of taxa, the CYP75 protein sequences from angiosperms are divided into two large groups, corresponding to *CYP75A* and *CYP75B* subfamily (Fig. 1), respectively. Within each of these two groups, CYP75 protein sequences were further diverged into 2 well-supported clades (monocot A, dicot A; monocot B, and dicot B; Fig. 1). The dicot A group can be split into two subclades, dicot A1 and dicot A2 (Fig. 1).

For Vitaceae, CYP75 protein sequences were clustered into three groups: Vitaceae A1, Vitaceae A2, and Vitaceae B (Fig. 1). Vitaceae A1 belongs to dicot A1 and Vitaceae A2 belongs to dicots A2 within the *CYP75A* subfamily. Vitaceae A1 is the largest group with 11 CYP75 protein sequence of *Vitis vinifera*. But other Vitaceae species have only one protein sequence of sequences based on our transcriptome data. *Cissus microcarpa, Cissus tuberosa*, and *Tetrastigma sichouense* all lack Vitaceae A1. Vitaceae A2 is close to a CYP75 protein sequences of *Camellia sinensis* (Fig. 1). *Cissus microcarpa*, *Cissus tuberosa*, *Cyphostemma maranguense*, *Pterisanthes eriopoda* and *Tetrastigma sichouense* lack CYP75 protein sequences, *i.e.,* Vitaceae A2. The Vitaceae B from dicots B belongs to *CYP75B* subfamily. All selected Vitaceae species except *Cissus microcarpa* possess CYP75 protein sequences corresponding to Vitaceae B (Fig. 1).

**Table 1:** Numbers of *CYP75* genes in 15 Vitaceae species.

**Genus**	**Species**	**Numbers of *CYP75* genes**
***Ampelocissus***	*Ampelocissus elegans*	3
***Ampelopsis***	*Ampelopsis arborea*	3
	*Ampelopsis cordata*	3
***Cayratia***	*Cayratia japonica*	3
***Cissus***	*Cissus microcarpa*	0
	*Cissus tuberosa*	1
***Cyphostemma***	*Cyphostemma sandersonii*	2
***Leea***	*Leea guineensis*	2
***Nothocissus***	*Nothocissus spicifera*	3
***Parthenocissus***	*Parthenocissus vitacea*	3
***Pterisanthes***	*pterisanthes_eriopoda*	3
***Rhoicissus***	*Rhoicissus digitate*	3
***Tetrastigma***	*Tetrastigma lawsonii*	1
***Vitis***	*Vitis rotundifolia*	3
	*Vitis tilifolia*	3

### The motif variation in *CYP75* gene

**Figure 2:**
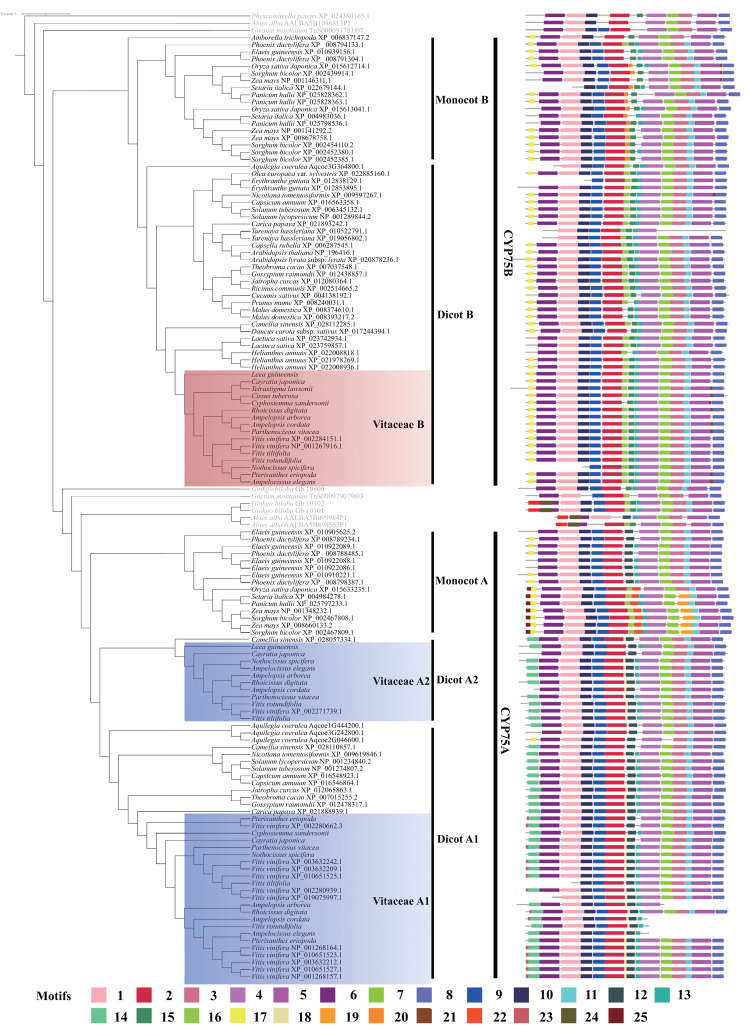
Motifs structure of *CYP75* gene family in Vitaceae and other angiosperm plants.

A total of 25 motifs were predicted in angiosperm CYP75 proteins (Fig. 2). CYP450s have four typical conserved motifs: the heme-binding region, PERF motif, K-helix region, and I-helix region (Crooks et al., 2004), which are found in our data (Fig. 2). In our analyses, motif 5 corresponds to the heme-binding region; motif 7 is corresponding to the k-helix region; motif 11 belongs to the PERF motif; and motif 4 corresponds to the I-helix region.

CYP75 protein sequences are conserved in most regions and the differences are mainly in the N terminal and the middle region (Fig. 2). Motif 1, 2, 3, 6, 8, 9, 10, 13 are distributed in most CYP75 proteins (Fig. 2). In the N terminal, CYP75 proteins from dicot A have a motif 14, which was replaced with motif 17 in dicot B, monocots B and monocots A (Fig. 2). In addition, motif 25 was only found in partial protein sequences of monocots A. Within the middle region, most protein sequences from dicot A have a motif 12, which was replaced by motifs 16 and 15 in dicot B, and replaced by motif 20 and motif 15 in monocot B, respectively (Fig. 2). Intriguingly, CYP75 protein sequences from monocot A have two different motifs composition, some include motif 12 and the other have motif 16 and 22. In the C terminal, most proteins have the same motifs, but motif 21 was detected only in a few CYP75B protein sequences and motif 19 was recovered in partial sequences within monocots A (Fig. 2).

CYP75 proteins from the three Vitaceae groups have a similar protein structure sharing many motifs (Fig. 2). Vitaceae A1 and Vitaceae A2 mostly share the same motifs, except that motif 23 is only found in the N terminal of protein sequences in Vitaceae A1 (Fig. 2). Compared with Vitaceae A1 and A2, motif 14 in Vitaceae B was replaced with motif 17 in protein sequences N terminal and motif 18, 12 in Vitaceae B were replaced by motifs 15, 16 in the middle part of protein sequences (Fig. 2).

### Purifying selection of *CYP75* gene family in angiosperms

We detected the variable selective pressures of the *CYP75* genes in the four groups: monocot A, monocot B, dicot A (dicot A1 and dicot A2) and dicot B (Table 2). The *ω* values under different branch models were estimated using PAML to understand the evolution of the four groups in angiosperms. The *ω*0 values were low (0.1127), suggesting strong purifying selection during *CYP75* gene family evolution. In monocot A, monocot B and dicot B, the branch model analysis with *CYP75* genes showed no significant difference between model 0 with one *ω* for all the sequences and model 2 with different d_N_/d_S_ ratios for the four groups (Table 2). But in dicot A, the model 0 and model 2 had a significant difference, indicating there was different selective pressure in this branch.

**Table 2:** Parameter estimates, lnL values and LRTs of codon substitution evolutionary analyses of selective patterns for *CYP75* gene family.

**Foreground branch**	**Model**	***ω* values**	**lnL**	***P***
Dicot A	One-ratio model 0	*ω*0 = 0.11276	−25629.435	
	(*ω*0= *ω*1 )			1.527e−03<0.05
	Two-ratio model 2	*ω*0 = 0.10644; *ω*1 = 0.13530	−25624.412	
	(*ω*0, *ω*1)			
Monocot A	One-ratio model 0	*ω*0 = 0.11276	−25629.435	
	(*ω*0= *ω*1 )			1.534e−01>0.05
	Two-ratio model 2	*ω*0 = 0.11069; *ω*1 = 0.12661	−25628.416	
	(*ω*0, *ω*1)			
Dicot B	One-ratio model 0	*ω*0 = 0.11276	−25629.435	
	(*ω*0= *ω*1 )			4.432e−01>0.05
	Two-ratio model 2	*ω*0 = 0.11496; *ω*1 = 0.10919	−25629.141	
	(*ω*0, *ω*1)			
Monocot B	One-ratio model 0	*ω*0 = 0.11276	−25629.435	
	(*ω*0= *ω*1 )			4.884e−01>0.05
	Two-ratio model 2	*ω*0 = 0.11387; *ω*1 = 0.10686	−25629.195	
	(*ω*0, *ω*1)			

## Discussion

Gene duplication provides the raw genetic material for the evolution of functional novelty and is considered to be a primary mechanism of functional diversification and expression divergence (Adams & Wendel, 2005; Soltis, Visger & Soltis, 2014). Our results support dividing the *CYP75* gene family into two groups in angiosperms (Fig. 1), corresponding to the *CYP75A* and *CYP75B* subfamilies (Tanaka, 2006; Zhang et al., 2019). Each group consists of both lineages of monocots and dicots, and a further duplication event is detected in dicots in the *CYP75A* subfamily (dicot A1, A2, Fig. 1).

In Vitaceae, analyses of phylogenetic relationships and protein structures support the classification of CYP75 protein sequences into three groups (Vitaceae A1, Vitaceae A2, and Vitaceae B, Figs. 1–2), in agreement with previous phylogenetic studies (Falginella et al., 2010). The Vitaceae A1 and A2 belong to the *CYP75A* subfamily and the Vitaceae B belongs to the *CYP75B* subfamily (Fig. 1). Most Vitaceae species have CYP75 protein sequences from each of three groups, but some species lack CYP75 protein sequences from some clades and no CYP75 protein sequences are detected in *Cissuss microcarpa* (Fig. 1). The observed lineage-specific gene loss events might have resulted from the lower quality of transcriptomes and/or the lack of deletion with incomplete sequences or highly diverged sequences in the phylogenetic analysis. But our result supports our preliminary exploration of the *CYP75* gene family from Vitaceae. The motif identification showed the three groups share many common motifs, indicating the CYP75 protein sequences are highly conserved. These common motifs are also conserved in other angiosperms (Fig. 2).

The strong conservation of the CYP75 protein sequences suggests that they are possibly maintained by functional constraints. Our selective pressure analysis also showed that the *ω*0 values of the *CYP75* gene family were 0.11276 in four angiosperm groups (Table 2). The results indicate the *CYP75* gene family might be under purifying selection in angiosperm plants, which is important for the highly conserved genes to be retained in the genome (Nei, 2007). The *CYP75* gene family is also under purifying selection from mosses to seed plants (Zhang et al., 2019). It seems that the *CYP75* gene family has been subject to pure selection pressure in the entire evolutionary history of plants, but a relatively relaxed selection pressure (*ω*1 = 0.13530) was found for the dicots in the *CYP75A* subfamily, which were split to dicot A1 and A2 after a gene duplication event (Table 2). The relaxed selective constraints resulted in elevated rates of evolution often occur after gene duplication events (Bielawski & Yang, 2001; Prince & Pickett, 2002; Wagner, 2002). For example, the Squamosa promoter binding protein (*SBP*)-box which encode plant-specific transcription factors can be divided into two groups, group I and group II (Zhang, Ling & Yi, 2015). The group II of *SBP-box* are under a relaxed purifying selection and can be divided into subgroup II-1 and subgroup II-2 after a gene duplication event (Zhang, Ling & Yi, 2015). The *CYP75A* genes in dicots might have had a functional divergence after the gene duplication.

Within Vitaceae A1, *Vitis vinifera* presents a high number of CYP75A proteins (Fig. 1). Previous research showed that many *CYP75A* genes copies reside in an array on the sixth chromosome in *Vitis vinifera*, which were initially duplicated through tandem gene duplication (Falginella et al., 2010). However, other species of Vitaceae do not have multiple protein sequences, including two other species of *Vitis* (Fig. 1). It seems that the high number of *CYP75A* proteins might occurred in *Vitis vinifera* after its split from the sister species and speciation might play an important role in the diversity of *CYP75A* genes in this species. Similar case was also found in other gene families. For example, terpene synthase (*TPS*) gene family in *Vitis vinifera* contain five *TPS* subfamilies (*TPS-a*, *-b*, *-c*, *-e*, *-g*) and the *TPS-a* form a large paralogous cluster which indicated post-speciation gene duplication events (Martin et al., 2010). A similar situation was also found in *Arabidopsis thaliana* (Aubourg, Lecharny & Bohlmann, 2002). However, the phylogenetic analysis shows that *Vitis vinifera* CYP75A proteins were not clustered into a monophyletic group in Vitaceae A1 (Fig. 1), which might suggest the complexity of gene duplication in grapes in different periods. The high CYP75A protein number of *Vitis vinifera* from many gene duplication events associated with active functions in *Vitis vinifera*. For example, the large number of the *CYP75A* genes play an important role in regulating the fruit color of different grapes cultivars, which were found to be temporally specialized for different developmental stages of berry ripening and also show an expression variation in different cultivars (Falginella et al., 2010). Among *CYP75A* genes from *Hordeum vulgare*, one copy is only expressed in aleurone layer and has a higher expression level in the blue aleurone than the uncolored aleurone and the other is expressed only in the barley grain and has a higher expression in the aleurone layer and pericarp in the green BW line compared colored ones (Vikhorev, Strygina & Khlestkina, 2019). The *CYP75A* also control the flower color, such as *Hf1* gene of petunia was expressed in both the limb and the tube of flowers and *Hf2* gene was only expressed in limb (Holton et al., 1993). It seems that different members of the *CYP75A* genes fulfill different roles in different tissues and times and are thus differentially retained in the various cultivars. In Vitaceae except for *Vitis vinifera*, functions of *CYP75A* genes need to be tested further.

The Vitaceae A2 and a CYP75A sequence (*CsF3′5′H*) from *Camellia sinensis* (Guo et al., 2019) were grouped together as dicot A2, distinct from dicot A1 within the CYP75A subfamily (Fig. 1). It showed that the gene duplication event might happen in the early divergence of dicots or at least before the split of Rosids and Asterids. Based on our current genomic sampling, CYP75A sequences of dicot A2 are only in taxa from the Vitaceae from Rosids and *Camellia sinensis* from Asterids (Fig. 1). The gene duplication had been studied in *Vitis vinifera*, which is in the ancestral lineage of eudicots at the time of *γ* triplication (Falginella et al., 2010; Zhang, Wen & Zimmer, 2016). *γ* triplication was coincident with rapid radiation of major lineages of core eudicots and appeared in the time before the origin and rapid radiation of core eudicots (Jiao et al., 2012). Our results suggested that many gene loss events occurred probably in most other dicots after the gene duplication in the *CYP75A* subfamily. In *Vitis vinifera*, the *CYP75A* gene (*F3′5′Hp*) from Vitaceae A2 maintained as a single-copy gene on chromosome 8 after the gene duplication, which has a high expression in all vegetative organs and a weak expression in fruits (Falginella et al., 2010). Similarly, the *CsF3′5′H* of *Camellia sinensis* can only catalyze flavonols and flavanonols and perhaps plays a key role in the 5′-hydroxylated of flavavonols (Guo et al., 2019). *Vitis vinifera* is an important fruit crop, which contains a large number of flavonoids (Conde et al., 2007). For *Camellia sinensis,* flavonoids are the most prominent metabolites and the content of about 20% flavan 3-ols in dry tea leaf (Punyasiri et al., 2004). We hypothesize that the dicot A2 group genes might represent a relict group of genes associated with production of abundant flavonoids, but more evidences from actual experiment of other Vitaceae taxa are needed to test this hypothesis.

The Vitaceae B belongs to the *CYP75B* subfamily, distinct from Vitaceae A1 and A2 from the *CYP75A* subfamily (Fig. 1). The motif difference between Vitaceae A and Vitaceae B is reflected in the N-terminal and middle part of the protein sequence (Fig. 2). The CYP75 protein sequences from Vitaceae B have a motif 17 in the N terminal, which was replaced with motif 14 in the Vitaceae A1 and Vitaceae A2 (Fig. 2). This difference also represents in other angiosperm species in the two branches of *CYP75A* and *CYP75B* subfamily (Fig. 2). The substrate specificities of flavonoid 3′-hydroxylases and flavonoid 3′, 5′-hydroxylases are defined near the N-terminal end (Seitz, Ameres & Forkmann, 2007). Motif 17 and motif 14 might be important to define the two types of proteins. In the middle part of protein sequences, CYP75 protein sequences from Vitaceae A1 and A2 include the motif 12, which was replaced with motif 15, 16 in Vitaceae B (Fig. 2). It suggested that the three motifs were also important features to distinguish two types of CYP75 proteins (Seitz, Ameres & Forkmann, 2007).

All Vitaceae species were found to have CYP75B proteins, except for *Cissuss microcarpa* (Fig. 1). The *CYP75B* gene of *F3′Ha* from *Vitis vinifera* is widely expressed in many organs (Falginella et al., 2010). There are many angiosperm plants retained with only *CYP75B* genes. For example, *Arabidopsis thaliana* only contains a *CYP75B* gene (*AtF3′H*), which has a high expression in siliques, providing precursors for tannin biosynthesis in the seed coat (Schoenbohm et al., 2000). The *CYP75B* gene of *sf’h1* from *Glycine max* is responsible for pubescence and seed coat color (Toda et al., 2002). In *Brassica napus*, the *CYP75B* genes play diverse roles in the synthesis of colored and colorless flavonoids in various organs to take on multiple functions. Furthermore, some *CYP75B* genes can function as *CYP75A* genes, such as in rice and Asteraceae species (Seitz et al., 2006; Lam, Liu & Lo, 2015). Therefore, it seems that *CYP75B* genes play important roles in plant growth and development and may also serve as a mutual compensation to the *CYP75A* subfamily in many angiosperms.

## Supplemental Information

10.7717/peerj.12174/supp-1 Supplemental Information 1 Information of *CYP75* genes from 36 plant species

## Additional Information and Declarations

### Data Availability

The following information was supplied regarding data availability: The Information of CYP75 genes from 36 plants species is available in the Supplemental File.
