# Supplementary material for: The expansion and diversity of the CYP75 gene family in Vitaceae
Source: PeerJ. 2021 Sep 15;9:e12174. doi: 10.7717/peerj.12174 (PMC8449539; doi:10.7717/peerj.12174)
Supplement: Supplemental Information 1 [file peerj-09-12174-s001.docx]

**Table S1.** Information of CYP75 genes from 36 plants species.

| **Species** | **Accessions** | **Source** |
| --- | --- | --- |
| **Bryophytes** |  |  |
| *Physcomitrella patens* | XP_24380165.1 | NCBI |
| **Gymnosperm** |  |  |
| *Abies alba* | AALBA5B099984P1 | (Mosca, Cruz et al. 2019) |
|  | AALBA5B1046813P1 | (Mosca, Cruz et al. 2020) |
|  | AALBA5B898565P1 | (Mosca, Cruz et al. 2021) |
| *Ginkgo biloba* | Gb_10102 | (Guan, Zhao et al. 2016) |
|  | Gb_10101 | (Guan, Zhao et al. 2017) |
|  | Gb_19800 | (Guan, Zhao et al. 2018) |
| *Gnetum montanum* | TnS000091781t07 | (Wan, Liu et al. 2018) |
|  | TnS000979079t03 | (Wan, Liu et al. 2019) |
| **Basal angiosperm** |  |  |
| *Amborella trichopoda* | XP_6837147.2 | NCBI |
| **Basal eudicots** |  |  |
| *Aquilegia coerulea* | Aqcoe1G444200.1 | Phytozome |
|  | Aqcoe2G046600.1 | Phytozome |
|  | Aqcoe3G242800.1 | Phytozome |
|  | Aqcoe3G364800.1 | Phytozome |
| **Asterids** |  |  |
| *Erythranthe guttata* | XP_12853895.1 | NCBI |
|  | XP_12838129.1 | NCBI |
| *Helianthus annuus* | XP_21978269.1 | NCBI |
|  | XP_22008936.1 | NCBI |
|  | XP_22008818.1 | NCBI |
| *Lactuca sativa* | XP_23742934.1 | NCBI |
|  | XP_23759857.1 | NCBI |
| *Solanum lycopersicum* | NP_1289844.2 | NCBI |
|  | NP_1234840.2 | NCBI |
| *Solanum tuberosum* | NP_1274807.2 | NCBI |
|  | XP_6345132.1 | NCBI |
| *Camellia sinensis* | XP_28112285.1 | NCBI |
|  | XP_28110857.1 | NCBI |
|  | XP_28057334.1 | NCBI |
| *Capsicum annuum* | XP_16563358.1 | NCBI |
|  | XP_16548923.1 | NCBI |
|  | XP_16546864.1 | NCBI |
| *Daucus carota* subsp. *sativus* | XP_17244394.1 | NCBI |
| *Nicotiana tomentosiformis* | XP_9597267.1 | NCBI |
|  | XP_9619846.1 | NCBI |
| *Olea europaea* var. *sylvestris* | XP_22885160.1 | NCBI |
| **Rosids** |  |  |
| *Malus domestica* | XP_8374610.1 | NCBI |
|  | XP_8393217.2 | NCBI |
| *Ricinus communis* | XP_2514665.2 | NCBI |
| *Arabidopsis lyrata* subsp. *lyrata* | XP_20878236.1 | NCBI |
| *Arabidopsis thaliana* | NP_196416.1 | NCBI |
| *Capsella rubella* | XP_6287545.1 | NCBI |
| *Carica papaya* | XP_21893242.1 | NCBI |
|  | XP_21888939.1 | NCBI |
| *Cucumis sativus* | XP_4138192.1 | NCBI |
| *Gossypium raimondii* | XP_12438857.1 | NCBI |
|  | XP_12478317.1 | NCBI |
| *Jatropha curcas* | XP_12080364.1 | NCBI |
|  | XP_12065863.1 | NCBI |
| *Prunus mume* | XP_8240031.1 | NCBI |
| *Tarenaya hassleriana* | XP_19056802.1 | NCBI |
|  | XP_10522740.1 | NCBI |
| *Theobroma cacao* | XP_7037548.1 | NCBI |
|  | XP_7015255.2 | NCBI |
| *Vitis vinifera* | NP_1267916.1 | NCBI |
|  | NP_1268164.1 | NCBI |
|  | NP_1268157.1 | NCBI |
|  | XP_2284151.1 | NCBI |
|  | XP_10651523.1 | NCBI |
|  | XP_3632212.1 | NCBI |
|  | XP_10651527.1 | NCBI |
|  | XP_2280939.1 | NCBI |
|  | XP_19075997.1 | NCBI |
|  | XP_10651525.1 | NCBI |
|  | XP_3632242.1 | NCBI |
|  | XP_3632209.1 | NCBI |
|  | XP_2271739.1 | NCBI |
|  | XP_2280662.3 | NCBI |
| **Monocots** |  |  |
| *Elaeis guineensis* | XP_10939156.1 | NCBI |
|  | XP_10910221.1 | NCBI |
|  | XP_10922086.1 | NCBI |
|  | XP_10922088.1 | NCBI |
|  | XP_10922089.1 | NCBI |
|  | XP_10905625.2 | NCBI |
| *Oryza sativa Japonica* | XP_15613041.1 | NCBI |
|  | XP_15612714.1 | NCBI |
|  | XP_15633235.1 | NCBI |
| *Panicum hallii* | XP_25798536.1 | NCBI |
|  | XP_25828363.1 | NCBI |
|  | XP_25828362.1 | NCBI |
|  | XP_25797233.1 | NCBI |
| *Phoenix dactylifera* | XP_8791304.1 | NCBI |
|  | XP_8798387.1 | NCBI |
|  | XP_8788485.1 | NCBI |
|  | XP_8789234.1 | NCBI |
|  | XP_8794133.1 | NCBI |
| *Setaria italica* | XP_4983036.1 | NCBI |
|  | XP_22679144.1 | NCBI |
|  | XP_4984278.1 | NCBI |
| *Sorghum bicolor* | XP_2454110.2 | NCBI |
|  | XP_2452380.1 | NCBI |
|  | XP_2452385.1 | NCBI |
|  | XP_2439914.1 | NCBI |
|  | XP_2467809.1 | NCBI |
|  | XP_2467808.1 | NCBI |
| *Zea mays* | NP_1141292.2 | NCBI |
|  | NP_1146311.1 | NCBI |
|  | NP_1348232.1 | NCBI |
|  | XP_8678758.1 | NCBI |
|  | XP_8660133.2 | NCBI |
